# Supplementary material for: Spillover of a Tobamovirus from the Australian Indigenous Flora to Invasive Weeds
Source: Viruses. 2022 Jul 29;14(8):1676. doi: 10.3390/v14081676 (PMC9413628; doi:10.3390/v14081676)
Supplement: Supplementary file 1 [file viruses-14-01676-s001.zip › viruses-1833813-supplementary.pdf]

Table S1. Wild Plants tested for the presence of yellow tailflower mild mottle virus, their collection sites in Perth metropolitan region (LY, Yanchep; LP, Ledge Point; LT, Lake Thetis; DT, Dirt Track; SH, Sandhills; BL, Bibra Lake; CR, Canning River; BTA and BTB, Bertram; MU: Murdoch; DR, Dixon Road; LM and LMB, Leeming), Western Australia.

No.	Species	Label of sample	RT-PCR test [a]
1	*Anthocercis illicifolia*	LY1	Negative
2	*A. illicifolia*	LY2	Negative
3	*A. illicifolia*	LY3	Negative
4	*A. illicifolia*	LY4	Negative
5	*A. illicifolia*	LY5	Negative
6	*A. illicifolia*	LY6	Negative
7	*A. illicifolia*	LY7	Negative
8	*A. illicifolia*	LY8	Positive
9	*A. illicifolia*	LY9	Negative
10	*A. illicifolia*	LY10	Negative
11	*A. illicifolia*	LY11	Positive
12	*A. illicifolia*	LY12	Positive
13	*A. illicifolia*	LY13	Negative
14	*A. illicifolia*	LY14	Negative
15	*A. illicifolia*	LY15	Positive
16	*A. illicifolia*	LY16	Positive
17	*A. illicifolia*	LY17	Positive
18	*A. illicifolia*	LY18	Negative
19	*A. illicifolia*	LY19	Negative
20	*A. illicifolia*	LY20	Negative
21	*A. illicifolia*	LY21	Negative
22	*A. illicifolia*	LY22	Negative
23	*A. illicifolia*	LY23	Negative
24	*A. illicifolia*	LY24	Positive
25	*A. illicifolia*	LY25	Positive
26	*A. illicifolia*	LY26	Negative
27	*A. illicifolia*	LP1	Positive
28	*A. illicifolia*	LP2	Positive
29	*A. illicifolia*	LP3	Positive
30	*A. illicifolia*	LP4	Positive
31	*A. illicifolia*	LP5	Positive
32	*A. illicifolia*	LT1	Negative
33	*A. illicifolia*	LT2	Negative
34	*A. illicifolia*	LT3	Negative
35	*A. illicifolia*	LT4	Negative
36	*A. illicifolia*	LT5	Negative
37	*A. illicifolia*	LT6	Positive
38	*A. illicifolia*	LT7	Positive

39	*A. illicifolia*	LT8	Positive
40	*A. illicifolia*	LT9	Positive
41	*A. illicifolia*	LT10	Positive
42	*A. illicifolia*	LT11	Positive
43	*A. illicifolia*	LT12	Positive
44	*A. illicifolia*	LT13	Positive
45	*A. illicifolia*	LT14	Positive
46	*A. illicifolia*	LT15	Positive
47	*A. illicifolia*	LT16	Negative
48	*A. illicifolia*	LT17	Negative
49	*A. illicifolia*	LT18	Positive
50	*A. illicifolia*	LT19	Positive
51	*A. illicifolia*	LT20	Positive
52	*A. illicifolia*	LT21	Negative
53	*Anthocercis littoria*	LP6	Negative
54	*A. littoria*	LP7	Negative
55	*A. littoria*	LP8	Negative
56	*A. littoria*	LP9	Negative
57	*A. littoria*	LP10	Negative
58	*A. littoria*	LP11	Negative
59	*A. littoria*	LP12	Negative
60	*A. littoria*	LP13	Negative
61	*A. littoria*	LP14	Negative
62	*A. littoria*	LP15	Negative
63	*A. littoria*	LP16	Negative
64	*A. littoria*	LP17	Negative
65	*A. littoria*	LP18	Negative
66	*A. littoria*	LP19	Negative
67	*A. littoria*	LP20	Negative
68	*A. littoria*	LP21	Negative
69	*A. littoria*	LP22	Negative
70	*A. littoria*	LP23	Negative
71	*A. littoria*	LP24	Negative
72	*A. littoria*	LP25	Negative
73	*A. littoria*	LP26	Negative
74	*A. littoria*	LP27	Negative
75	*A. littoria*	LP28	Negative
76	*A. littoria*	LP29	Negative
77	*A. littoria*	LP30	Negative
78	*A. littoria*	LP31	Negative
79	*A. littoria*	LP32	Negative
80	*A. littoria*	LP33	Negative
81	*A. littoria*	DT1	Negative
82	*A. littoria*	DT2	Positive

83	*A. littoria*	DT3	Positive
84	*A. littoria*	DT4	Positive
85	*A. littoria*	DT5	Negative
86	*A. littoria*	SH1	Positive
87	*A. littoria*	SH2	Positive
88	*A. littoria*	SH3	Negative
89	*A. littoria*	SH4	Negative
90	*A. littoria*	SH5	Negative
91	*A. littoria*	SH6	Positive
92	*A. littoria*	SH7	Positive
93	*A. littoria*	SH8	Positive
94	*A. littoria*	SH9	Positive
95	*A. littoria*	SH10	Negative
96	*A. littoria*	SH11	Positive
97	*A. littoria*	SH12	Positive
98	*A. littoria*	SH13	Negative
99	*A. littoria*	SH14	Positive
100	*A. littoria*	SH15	Positive
101	*A. littoria*	SH16	Negative
102	*A. littoria*	SH17	Negative
103	*A. littoria*	SH18	Negative
104	*A. littoria*	SH19	Negative
105	*A. littoria*	SH20	Positive
106	*A. littoria*	SH21	Positive
107	*A. littoria*	SH22	Negative
108	*A. littoria*	SH23	Negative
109	*Physalis peruviana*	LY27	Negative
110	*P. peruviana*	LY28	Positive
111	*P. peruviana*	LY29	Positive
112	*P. peruviana*	LY30	Negative
113	*P. peruviana*	LY31	Positive
114	*P. peruviana*	LY32	Positive
115	*P. peruviana*	LY33	Positive
116	*P. peruviana*	LY60	Positive
117	*Solanum lycopersicum*	BL12	Negative
118	*S. nigrum*	LY34	Positive
119	*S. nigrum*	LY35	Negative
120	*S. nigrum*	LY36	Positive
121	*S. nigrum*	LY37	Positive
122	*S. nigrum*	LY38	Negative
123	*S. nigrum*	LY39	Negative
124	*S. nigrum*	LY40	Positive
125	*S. nigrum*	LY41	Positive
126	*S. nigrum*	LY42	Positive

127	*S. nigrum*	LY43	Positive
128	*S. nigrum*	LY44	Negative
129	*S. nigrum*	LY45	Negative
130	*S. nigrum*	LY46	Positive
131	*S. nigrum*	LY47	Negative
132	*S. nigrum*	LY48	Positive
133	*S. nigrum*	LY49	Negative
134	*S. nigrum*	LY50	Positive
135	*S. nigrum*	LY51	Positive
136	*S. nigrum*	LY52	Positive
137	*S. nigrum*	LY53	Negative
138	*S. nigrum*	LY54	Negative
139	*S. nigrum*	LY55	Negative
140	*S. nigrum*	LY56	Positive
141	*S. nigrum*	LY57	Positive
142	*S. nigrum*	LY58	Negative
143	*S. nigrum*	LY59	Positive
144	*S. nigrum*	BL1	Negative
145	*S. nigrum*	BL2	Negative
146	*S. nigrum*	BL3	Negative
147	*S. nigrum*	BL4	Negative
148	*S. nigrum*	BL5	Positive
149	*S. nigrum*	BL6	Negative
150	*S. nigrum*	BL7	Negative
151	*S. nigrum*	BL8	Positive
152	*S. nigrum*	BL9	Negative
153	*S. nigrum*	BL10	Negative
154	*S. nigrum*	BL11	Negative
155	*S. nigrum*	CR1	Negative
156	*S. nigrum*	CR2	Negative
157	*S. nigrum*	CR3	Negative
158	*S. nigrum*	CR4	Negative
159	*S. nigrum*	BTA1	Positive
160	*S. nigrum*	BTA2	Positive
161	*S. nigrum*	BTA3	Negative
162	*S. nigrum*	BTA4	Positive
163	*S. nigrum*	BTA5	Positive
164	*S. nigrum*	BTA6	Positive
165	*S. nigrum*	BTA7	Positive
166	*S. nigrum*	BTA8	Negative
167	*S. nigrum*	BTA9	Negative
168	*S. nigrum*	BTA10	Positive
169	*S. nigrum*	BTB1	Positive
170	*S. nigrum*	BTB2	Negative

171	*S. nigrum*	BTB3	Positive
172	*S. nigrum*	BTB4	Negative
173	*S. nigrum*	BTB5	Positive
174	*S. nigrum*	BTB6	Positive
175	*S. nigrum*	BTB7	Negative
176	*S. nigrum*	MU1	Negative
177	*S. nigrum*	MU2	Negative
178	*S. nigrum*	MU3	Negative
179	*S. nigrum*	MU4	Negative
180	*S. nigrum*	DR1	Negative
181	*S. nigrum*	DR2	Positive
182	*S. nigrum*	DR3	Positive
183	*S. nigrum*	DR4	Positive
184	*S. nigrum*	DR5	Negative
185	*S. nigrum*	DR6	Positive
186	*S. nigrum*	LM1	Positive
187	*S. nigrum*	LM2	Negative
188	*S. nigrum*	LM3	Positive
189	*S. nigrum*	LM4	Positive
190	*S. nigrum*	LM5	Positive
191	*S. nigrum*	LM6	Positive
192	*S. nigrum*	LM7	Negative
193	*S. nigrum*	LM8	Negative
194	*S. nigrum*	LM9	Positive
195	*S. nigrum*	LM10	Positive
196	*S. nigrum*	LM11	Positive
197	*S. nigrum*	LM12	Negative
198	*S. nigrum*	LM13	Negative
199	*S. nigrum*	LM14	Negative
200	*S. nigrum*	LMB1	Positive
201	*S. nigrum*	LMB2	Positive
202	*S. nigrum*	LMB3	Positive
203	*S. nigrum*	LMB4	Negative
204	*S. nigrum*	LMB5	Negative
205	*S. nigrum*	LMB6	Positive
206	*S. nigrum*	LMB7	Positive
207	*S. nigrum*	LMB8	Negative
208	*S. nigrum*	LMB9	Negative
209	*S. nigrum*	LMB10	Negative

[a] Positive or negative for the presence of YTMMV using virus-specific primers YTMMVCPF: 5′-GCTTAAAGAGCGAATTGATGAG and YTMMVCPR: 5′-CCATTGTAGTCTTGCACAGCAC that amplified a fragment of the viral coat protein.

Table S2. Host species and original location of yellow tailflower mild mottle virus (YTMMV) isolates used in this study. Genbank accession codes are of partial (356 nt) viral CP genes.

Species	Location	YTMMV isolate	Genbank accession
Solanum nigrum	Bertram	ST-BTA1	ON733090
S. nigrum	Bertram	ST-BTA2	ON733091
S. nigrum	Bertram	BTA4	ON733069
S. nigrum	Bertram	BTA5	ON733070
S. nigrum	Bertram	BTA6	ON733071
S. nigrum	Bertram	BTB1	ON733072
S. nigrum	Bertram	BTB3	ON733073
S. nigrum	Bertram	BTB6	ON733074
S. nigrum	Bibra Lake	BL5	ON733067
S. nigrum	Bibra Lake	BL8	ON733068
Anthcercis littoria	Cervantes Dirt Track	DT3	ON733048
A. littoria	Cervantes Dirt Track	DT4	ON733049
A. littoria	Cervantes Sandhills	SH1	ON733050
A. littoria	Cervantes Sandhills	SH2	ON733051
A. littoria	Cervantes Sandhills	SH7	ON733052
A. littoria	Cervantes Sandhills	SH8	ON733053
A. littoria	Cervantes Sandhills	SH11	ON733054
A. littoria	Cervantes Sandhills	SH12	ON733055
A. littoria	Cervantes Sandhills	SH14	ON733056
A. littoria	Cervantes Sandhills	SH15	ON733057
S. nigrum	Dixon road	ST-DR2	ON733092
S. nigrum	Dixon road	DR3	ON733075
S. nigrum	Dixon road	DR6	ON733076
Anthocercis illicifolia	Lake Thetis	LT6	ON733045
A. illicifolia	Lake Thetis	LT7	ON733046
A. illicifolia	Lake Thetis	LT8	ON733047
A. illicifolia	Ledge Point	LP1	ON733042
A. illicifolia	Ledge Point	LP2	ON733043
A. illicifolia	Ledge Point	LP4	ON733044
S. nigrum	Leeming	LM3	ON733077
S. nigrum	Leeming	LM4	ON733078
S. nigrum	Leeming	LM6	ON733079
S. nigrum	Leeming	LM9	ON733080
S. nigrum	Leeming	LM10	ON733081
S. nigrum	Leeming	LMB1	ON733082
S. nigrum	Leeming	LMB3	ON733083
S. nigrum	Leeming	LMB6	ON733084
S. nigrum	Leeming	LMB7	ON733085
S. nigrum	Leeming	LMB2	ON733086

A. illicifolia	Yanchep	LY8	ON733038
A. illicifolia	Yanchep	LY11	ON733039
A. illicifolia	Yanchep	LY12	ON733040
A. illicifolia	Yanchep	LY15	ON733041
Physalis peruviana	Yanchep	LY28	ON733058
P. peruviana	Yanchep	LY31	ON733059
P. peruviana	Yanchep	LY60	ON733060
S. nigrum	Yanchep	LY34	ON733061
S. nigrum	Yanchep	LY37	ON733062
S. nigrum	Yanchep	LY41	ON733063
S. nigrum	Yanchep	LY43	ON733064
S. nigrum	Yanchep	LY51	ON733065
S. nigrum	Yanchep	LY56	ON733066

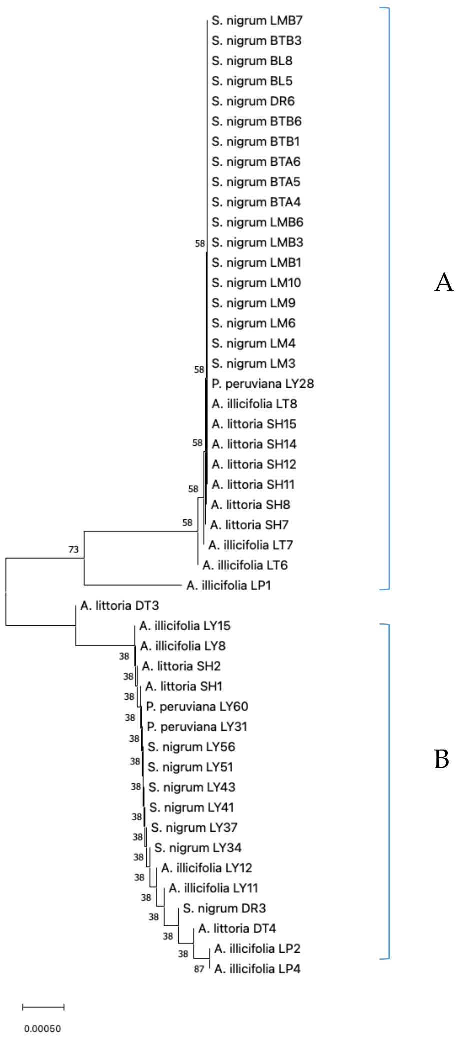

Figure S1. Pairwise comparison of partial CP sequences (356 nt) of 48 YTMMV isolates from wild plants, indigenous and exotic, inferred by the Neighbor-Joining method. Host species and virus isolate names are given (GenBank accession codes given in Table S1). Virus isolate names include the site from which they were collected: LY, Yanchep; LP, Ledge Point; LT, Lake Thetis; DT, Dirt Track (Cervantes region); SH, Sandhills (Cervantes); BL, Bibra lake; CR, Canning River; BTA and BTB, Bertram; MU, Murdoch; DR, Dixon Road; LM and LMB, Leeming. The isolates fell into two main groups (A and B) as marked. The tree was drawn to scale, with branch lengths in the same units as those of the evolutionary distances used to infer the phylogenetic tree. The units of the number of base substitutions per site.
